# Taxonomic and Enzymatic Characterization of *Flocculibacter collagenilyticus* gen. nov., sp. nov., a Novel Gammaproteobacterium With High Collagenase Production

**DOI:** 10.3389/fmicb.2021.621161

**Published:** 2021-03-09

**Authors:** Jian Li, Jun-Hui Cheng, Zhao-Jie Teng, Zhong-Zhi Sun, Xiao-Yan He, Peng Wang, Mei Shi, Xiao-Yan Song, Xiu-Lan Chen, Yu-Zhong Zhang, Xinmin Tian, Xi-Ying Zhang

**Affiliations:** ^1^College of Life Science and Technology, Xinjiang University, Urumqi, China; ^2^State Key Laboratory of Microbial Technology, Institute of Marine Science and Technology, Marine Biotechnology Research Center, Shandong University, Qingdao, China; ^3^Laboratory for Marine Biology and Biotechnology, Pilot National Laboratory for Marine Science and Technology, Qingdao, China; ^4^College of Marine Life Sciences, and Frontiers Science Center for Deep Ocean Multispheres and Earth System, Ocean University of China, Qingdao, China

**Keywords:** *Flocculibacter* gen. nov., *Pseudoalteromonadaceae*, collagenase, collagenase-secreting bacteria, the MEROPS S8 family proteases

## Abstract

Collagens from marine animals are an important component of marine organic nitrogen. Collagenase-producing bacteria and their collagenases play important roles in collagen degradation and organic nitrogen recycling in the ocean. However, only a few collagenase-producing marine bacteria have been so far discovered. Here, we reported the isolation and characterization of a collagenase-secreting bacterium, designated strain SM1988^T^, isolated from a green alga *Codium fragile* sample. Strain SM1988^T^ is a Gram-negative, aerobic, oxidase-, and catalase-positive, unipolar flagellated, and rod-shaped bacterium capable of hydrolyzing casein, gelatin and collagens. Phylogenetic analysis revealed that strain SM1988^T^ formed a distinct phylogenetic lineage along with known genera within the family *Pseudoalteromonadaceae*, with 16S rRNA gene sequence similarity being less than 93.3% to all known species in the family. Based on the phylogenetic, genomic, chemotaxonomic and phenotypic data, strain SM1988^T^ was considered to represent a novel species in a novel genus in the family *Pseudoalteromonadaceae*, for which the name *Flocculibacter collagenilyticus* gen. nov., sp. nov. is proposed, with the type strain being SM1988^T^ (= MCCC 1K04279^T^ = KCTC 72761^T^). Strain SM1988^T^ showed a high production of extracellular collagenases, which had high activity against both bovine collagen and codfish collagen. Biochemical tests combined with genome and secretome analyses indicated that the collagenases secreted by strain SM1988^T^ are serine proteases from the MEROPS S8 family. These data suggest that strain SM1988^T^ acts as an important player in marine collagen degradation and recycling and may have a promising potential in collagen resource utilization.

## Introduction

The family *Pseudoalteromonadaceae* within the order *Alteromonadales* of the class *Gammaproteobacteria* was proposed by Ivanova et al. (2004). The genus now comprises 4 genera (56 species): *Algicola* (2 species), *Pseudoalteromonas* (the type genus, 49 species), *Psychrosphaera* (4 species), and *Salsuginimonas* (1 species) (Parte et al., 2020)^1^. Species belonging to the family *Pseudoalteromonadaceae* are Gram-negative, rod-shaped, aerobic or facultatively anaerobic, chemoorganotrophic bacteria, having C_16:0_, C_16:1_ ω7*c*, and C_18:1_*ω7c* as the major cellular fatty acids and Q-8 as the major isoprenoid quinone (Ivanova et al., 2004). Members of this family have been isolated from diverse environments, including seawater, sediments, sea ice, marine plants, and animals (Lau et al., 2005; Al Khudary et al., 2008; Park et al., 2010; Lee et al., 2014; Ying et al., 2016; Sheu et al., 2017; Navarro-Torre et al., 2020). They play important ecological roles in marine environments through the production of extracellular proteases and a myriad of metabolites (Holmström et al., 1998; Ivanova et al., 2004; Kalinovskaya et al., 2004; Bowman, 2007; Chen et al., 2007; Evans et al., 2007; Qin et al., 2007; Schweder et al., 2008; He et al., 2012; Ran et al., 2014).

Collagen is the most abundant protein in marine animals, which widely distributes in various tissues such as skin, muscle, bone, tendons, teeth, and blood vessels (Bhattacharjee and Bansal, 2005; Zhao et al., 2008; Zhang et al., 2015). Due to its abundance, collagen is an important component of marine organic nitrogen, and therefore, the degradation of collagen is important for marine organic nitrogen recycling. However, because of its complex and special structure, collagen is difficult for common proteases to degrade, and only a few types of proteases from bacteria and mammals are known to be able to degrade collagen (Zhang et al., 2015). Marine collagenase-producing bacteria are an important force to drive marine collagen degradation and recycling (Ran et al., 2014). However, so far, only a few collagenase-producing marine bacteria have been reported, including *Pseudomonas marinoglutinosa* strain 7-246-6 (Kazunori et al., 2014), *Alkalimonas collagenimarina* AC40^T^ (Kurata et al., 2007), *Pseudoalteromonas* sp. SM9913 (Zhao et al., 2008), *Myroides profundi* D25^T^ (Ran et al., 2014), and *Pseudoalteromonas* sp. SJN2 (Yang et al., 2017). Therefore, identifying more marine collagenase-producing bacteria and their extracellular collagenases is essential for comprehensive understanding of marine collagen degradation and recycling. In addition, bacterial collagenases are potential tools for the preparation of collagen oligopeptides that are widely used in medicine, cosmetics and functional food (Chen et al., 2017).

The intertidal zone is a fluctuated environment where high bacterial diversity, abundance, and activities are usually found. In this study, a whitish colony-forming bacterial strain with high collagenase production, designated as SM1988^T^, was isolated from a green algal sample collected from the intertidal zone of Qingdao City, China. Strain SM1988^T^ is prominent by its low 16S rRNA gene sequence similarity (<94%) to known species in the class *Gammaproteobacteria* and its strong ability to secrete collagenases. In this study, the exact taxonomic position of strain SM1988^T^ was determined by using a polyphasic approach, and the enzymatic characteristics of the proteases secreted by strain SM1988^T^ were assayed through biochemical tests combined with genome and secretome analyses.

## Materials and Methods

### Sample Collection, and Isolation, and Cultivation of Bacterial Strains

The green algal sample of *Codium fragile* was collected from the intertidal zones (36°5′21″ N, 120°29′42″ E) in Qingdao City, China, on October 25, 2018. The sample was placed into a sterile plastic bag and transported to the laboratory under a low-temperature condition (4–6°C). In the laboratory, the algal sample was placed into a flask containing 10 mL sterile artificial seawater (3% Sigma sea salts, w/v) and 20 glass beads, which was subsequently shaken at 25°C for 10 min. The suspension was 10-fold serially diluted using sterile artificial seawater. An aliquot (100 μl) of each dilution was then spread on the TYS agar [0.5% tryptone (Oxoid, United Kingdom), 0.1% yeast extract (Oxoid, United Kingdom), 1.5% agar and artificial seawater] plates and incubated at 25°C for 5 days. Morphologically different colonies were picked and streaked on the TYS agar plates several times to acquire pure cultures. Strains purified were grown in TYS broth (0.5% tryptone, 0.1% yeast extract, and artificial seawater) and preserved by supplementing 18% (v/v) glycerol at −80°C. Strain SM1988^T^ was routinely cultured in TYS broth or on TYS agar at 25°C. *Pseudoalteromonas mariniglutinosa* DSM 15203^T^, *Pseudoalteromonas haloplanktis* MCCC 1A06496^T^, and *Psychrosphaera haliotis* JCM 16340^T^ were purchased from German Collection of Microorganisms and Cell Cultures (DSMZ), Marine Culture Collection of China (MCCC), and Japan Collection of Microorganisms (JCM), respectively, and were used as the reference strains in some phenotypic and chemotaxonomic tests.

### Sequencing and Analyses of the 16S rRNA Gene and the Whole Genome

Genomic DNA of strain SM1988^T^ was extracted by using a bacterial genomic DNA isolation kit (BioTeke, China) according to the manual instruction. The 16S rRNA gene was amplified using the universal primers 27F and 1492R by PCR (Lane, 1991), and sequenced using an automatic ABI Prism 3730XL sequencer (Applied Biosystems, United States). The obtained 16S rRNA gene sequence (GenBank accession number: MN606291) was compared with those in EzBioCloud databases (Yoon et al., 2017)^2^ to find phylogenetic neighbors. Sequences were aligned using the ClustalW program (Thompson et al., 1994) implemented in MEGA X (Kumar et al., 2018). The phylogenetic trees were constructed using the NJ (neighbor-joining) (Saitou and Nei, 1987), ML (maximum-likelihood) (Felsenstein, 1981), and MP (maximum-parsimony) (Fitch, 1971) methods. Evolutionary distances were calculated using Kimura’s two-parameter model (Kimura, 1980). Bootstrap analysis was performed based on 1000 replicates.

The whole-genome sequencing was carried out on a PacBio Sequel platform (Tianjin Novogene, China), and *de novo* genome assembly was performed using the HGAP 4 in SMRT Link (version 6.0.0.47841). rRNA and tRNA genes were identified by barrnap^3^ and tRNAscan-SE (Chan and Lowe, 2019), respectively. The functional gene annotations were performed by the Rapid Annotations using Subsystems Technology (RAST) server (Aziz et al., 2008). The assignment of predicted proteases to different families was performed using the peptidase database MEROPS (Rawlings et al., 2018)^4^. Signal peptides were predicted using the SignalP 5.0 server (Almagro Armenteros et al., 2019). The *in silico* DNA–DNA hybridization (*is*DDH) values were calculated using the Genome-to-Genome Distance Calculator 2.1 (Meier-Kolthoff et al., 2013)^5^. Average nucleotide identity (ANI) values were calculated using the JSpeciesWS (Richter et al., 2016)^6^. The average amino acid identity (AAI) values were calculated by the online AAI calculator developed in Environmental Microbial Genomics Laboratory (Rodriguez-R and Konstantinidis, 2014)^7^. The percentage of conserved proteins (POCP) values were calculated as described in Qin et al. (2014). The whole genome sequence of strain SM1988^T^ was deposited in GenBank under the accession number CP059888.

### Morphological, Physiological, and Biochemical Characterization

Colony morphology of strain SM1988^T^ was observed after incubation on TYS agar plates at 25°C for 5 days. Cellular morphology and the flagella were observed by Atomic Force Microscopy (AFM, Bruker AXS, Germany) and Transmission Electron Microscopy (TEM, Tecnai G2 F20, FEI, United States) with cells cultivated in TYS broth at 25°C for 10 h. Gram staining was performed according to Hucker’s method (Murray et al., 1994). The temperature (4, 10, 15, 20, 25, 30, and 37°C) and pH [5-10 at increments of 1.0 pH unit, buffered with 50 mM MES (pH 5–6), MOPS (pH 6.5–7.0), Tris (pH 7.5–8.5), and CHES (pH 9.0–10)] ranges for growth were examined in TYS broth. The NaCl tolerance was examined in the modified TYS broth where the artificial seawater was replaced with distilled water containing appropriate concentration of NaCl (0, 1, 2, 2.5, 3, 4, 5, 6, 7, 8, 9, and 10%, w/v). Growth under anaerobic condition was checked in TYS broth supplemented with potassium nitrate (0.1%, w/v), sodium sulfide (0.05%, w/v), and cysteine hydrochloride (0.05%, w/v) in Hungate tubes at 25°C for 30 days. Catalase activity was examined by observing whether bubbles were produced when pouring 3% H_2_O_2_ (v/v) on the colonies. Oxidase activity was examined using the Merck Bactident-Oxidase test strips (Merck, Germany). Hydrolysis of starch (1%, w/v), Tweens 20, 40, 60, and 80 (1%, v/v), casein (1%, w/v), gelatin (2%, w/v), elastin (0.5%, w/v), and aesculin (0.01%, w/v) was tested on TYS agar at 25°C (Smibert and Krieg, 1994). Antibiotic sensitivity tests were performed on TYS agar using discs (Oxoid, United Kingdom) impregnated with various antibiotics (per disc) including: ampicillin (10 μg/disc), penicillin G (10 μg/disc), vancomycin (30 μg/disc), chloramphenicol (30 μg/disc), carbenicillin (300 units/disc), polymyxin B (300 units/disc), novobiocin (5 μg/disc), erythromycin (15 μg/disc), gentamicin (10 μg/disc), tetracycline (30 μg/disc), colistin sulfate (10 μg/disc), oleandomycin (15 μg/disc), streptomycin (10 μg/disc), neomycin (10 μg/disc), cephalexin (30 μg/disc), and kanamycin (30 μg/disc). Enzymatic activities and other biochemical characteristics were determined using API 20NE and API ZYM (bioMérieux, France) strips according to the manufacturer’s instructions except that cells were suspended in 2.5% NaCl solution before inoculated into the strips.

### Chemotaxonomic Analysis

For the analysis of cellular fatty acids and polar lipids, strain SM1988^T^ and reference strains were cultivated in TYS broth at 25°C for 3 days. Extraction, saponification, and methylation of fatty acids were performed following the protocol of the Sherlock Microbial Identification System (MIS, MIDI Inc., United States), and fatty acid methyl esters (FAMEs) were analyzed by using GC (Hewlett Packard 6890, United States) and Sherlock MIS software (version 6.3). The polar lipids were extracted following the method described by Komagata and Suzuki (1987) and analyzed using two dimensional thin layer chromatography (TLC) with different spraying reagents including ethanolic molybdophosphoric acid [10% (w/v), total lipids], Zinzadze reagent (phospholipids) and ninhydrin (amino lipids) to visualize and identify lipid spots on the TLC plates (Collins and Jones, 1980). Respiratory quinones were extracted according to the method described by Komagata and Suzuki (1987) and were analyzed by HPLC-MS.

### Enzymatic Activity and Property Assays

Strain SM1988^T^ was cultured in the TYS broth medium at 25°C for 6 days. The culture was collected, and centrifuged (4,656 *g*, 10 min) at 4°C. The supernatant obtained was properly diluted 0–100 times with 50 mM Tris-HCl buffer (pH 8.0) prior to the enzymatic activity and property assays of the extracellular proteases of strain SM1988^T^. Protein concentration of the supernatant was determined by the bicinchoninic acid (BCA) method using a BCA protein assay kit (Thermo, United States) with bovine serum albumin (BSA) as the standard. The activities of the extracellular proteases toward bovine bone collagen, bovine tendon collagen and gelatin at 30 and 55°C and toward codfish collagen at 15 and 55°C were measured by the method provided by Worthington Biochemical Co. (Zhao et al., 2008). One unit of enzyme activity was defined as the amount of enzyme that released 1 nmol of L-leucine/h from collagen or gelatin. The caseinolytic activity was determined at 30 and 55°C using the method described by He et al. (2004). One unit of enzyme activity was defined as the amount of enzyme required to liberate 1 μg tyrosine per minute. The elastolytic activity at 30 and 55°C was determined using the method described by Chen et al. (2009). One unit of enzyme activity was defined as the amount of enzyme that caused an increase of 0.01 OD_590_/min. The enzyme activity against azocoll collagen at 30 and 55°C was measured according to the method of Abraham and Breuil (1996). One unit of enzyme activity was defined as the amount of enzyme that caused an increase of 0.1 OD_520_/min. Casein (from bovine milk), gelatin (from cold water fish skin), elastin-orcein, and azo dye-impregnated collagen (Azocoll) were purchased from Sigma (United States), bovine tendon collagen from Worthington (United States), and bovine bone collagen from a local market. Codfish collagen was extracted with the method described by Nagai et al. (1999).

To determine the extracellular protease production at different temperatures, strain SM1988^T^ was cultured at 15, 20, 25, and 30°C for 8 days, and the protease activity in the culture was measured at 55°C daily with bovine bone collagen as substrate. The optimal temperature for the extracellular proteases was determined by measuring the protease activity toward bovine bone collagen in 50 mM Tris-HCl buffer (pH 8.0) at 30, 40, 50, 55, 60, 70, and 80°C. To determine the optimal pH for the extracellular proteases, the culture supernatant was properly diluted 50 mM of the following buffers: citric acid-sodium citrate buffer (pH 4.0–6.0), phosphate buffer (pH 6.0–8.0), Tris-HCl buffer (pH 8.0–9.0), and Na_2_CO_3_-NaHCO_3_ buffer (pH 9.0–11.0), and then the protease activity toward bovine bone collagen was measured at 55°C. For the inhibitory experiment, the culture supernatant was 100 times diluted, and then incubated at 4°C for 1 h with 2 or 10 mM of the following inhibitors: phenylmethylsulfonyl fluoride (PMSF), ethylene-diamine tetraacetic acid (EDTA), ethylene glycol tetraacetic acid (EGTA), or *o*-phenanthroline (*o*-P). After incubation, the residue protease activity toward bovine bone collagen was measured at 55°C, pH 8.0. Gelatin and casein zymography were performed according to the method of Heussen and Dowdle (1980). The concentration of gelatin or casein in the gel was 1% (w/v).

### Secretome Analysis

Strain SM1988^T^ was cultivated in the TYS broth medium at 25°C for 6 days, and then 5 mL of the culture was collected and centrifuged. The secreted proteins in the supernatant were precipitated with 10% trichloroacetic acid. The protein pellets were suspended in 100% ice-cold acetone by vortexing for 30 min after which acetone was removed by centrifugation and air drying. The air-dried proteins were dissolved in a denaturing buffer (4% SDS, 100 mM Tris-HCl, pH 7.6), reduced with 10 mM DTT at 37°C for 1 h and then the SDS buffer was replaced with the UA buffer (8 M Urea, 100 mM Tris-HCl, pH 8.5) through a Microcon YM-30 centrifugal filter (Millipore, United States). After reduction, the proteins were alkylated with 55 mM iodoacetamide in the UA buffer at room temperature for 15 min in dark, and centrifuged at 12,000 *g* for 10 min to remove the UA buffer. Then, 50 μg proteins was dissolved in 50 μL of 1 mM triethylammonium hydrogen carbonate (TEAB) buffer (pH 8.0), and digested with 1 μg sequencing grade trypsin at 37°C overnight. The resultant peptides were desalted by StageTip and analyzed by LC-MS/MS at the Institute of Genetics and Development Biology, Chinese Academy of Sciences.

For LC-MS/MS analyses, 1 μg of the peptides were resuspended in 0.1% formic acid and analyzed by LTQ Orbitrap Elite mass spectrometer (Thermo Fisher Scientific, United States) coupled online to an Easy-nLC 1000 (Thermo Fisher Scientific, United States) in the data-dependent mode. The peptides were separated by reverse phase LC with a 150 μm (ID) × 250 mm (length) analytical column packed with C18 particles of 1.9 μm diameter in a 90 min gradient. All MS measurements were performed in the positive ion mode. Precursor ions were measured in the Orbitrap analyzer (Thermo Fisher Scientific, United States) at 240,000 resolution (at 400 m/z) and a target value of 10^6^ ions. The twenty most intense ions from each MS scan were isolated, fragmented and measured in the linear ion trap. The CID normalized collision energy was set to 35. The data were analyzed by Thermo Scientific Proteome Discoverer^TM^ software version 1.4, which used the reverse protein sequences of SM1988^T^ as a decoy database. The proteome sequences of SM1988^T^ from whole-genome sequences were used for the database searching. The false discovery rates (FDR) for peptide identifications were set to 0.01, and the mass tolerances used for MS1 and MS2 were 10 ppm and 0.5 Da, respectively. Methionine oxidation was included in the search as the variable modifications. Cysteine carbamidomethylation was set as stable modifications. The secretome data was uploaded to PRIDE under the accession number PXD023431.

The MOROPS S8 proteases in the secretome of strain SM1988^T^, deseasin MCP-01 (ABD14413) from *Pseudoalteromonas* sp. SM9913 (Chen et al., 2007), the collagenolytic protease (BAF30978) from *Geobacillus* sp. MO-1 (Itoi et al., 2006), and myroicolsin from *Myroides profundi* D25 (Ran et al., 2014) were aligned by ClustalW in the software MEGA X (Kumar et al., 2018) and the alignment was displayed using ESPript 3.0 (Robert and Gouet, 2014)^8^. The conserved motifs were predicted by MEME (Bailey et al., 2009).

## Results

### Analysis of the 16S rRNA Gene and Genomic Sequences

**FIGURE 1:**
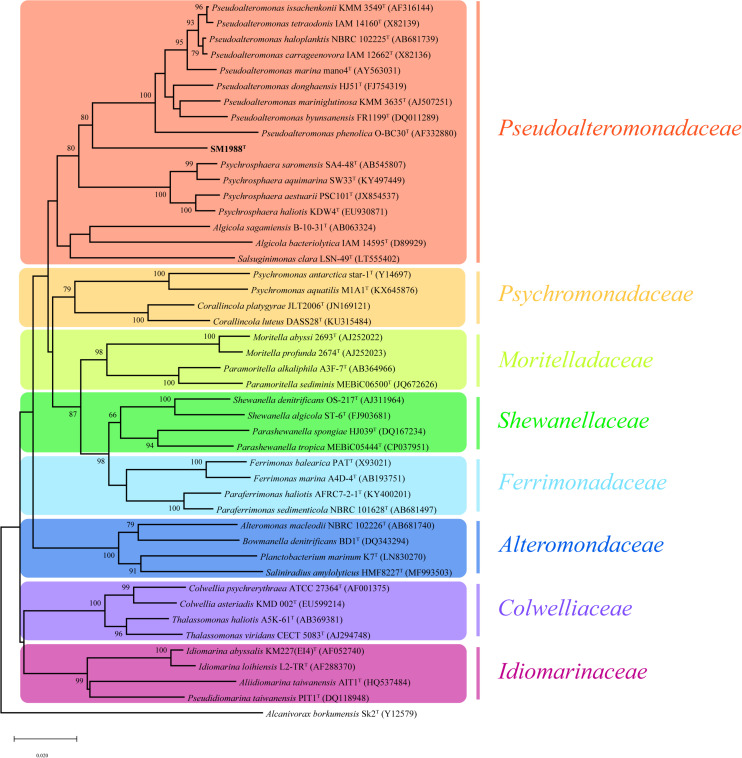
Neighbor-joining (NJ) phylogenetic tree based on 16S rRNA gene sequences showing the positions of strain SM1988^T^ (in bold) and related taxa in the class *Gammaproteobacteria*. Bootstrap values (>70%) based on 1,000 replicates were presented at nodes. *Alcanivorax borkumensis* Sk2^T^ was used as the outgroup. Bar, 0.02 substitutions per nucleotide position.

Strain SM1988^T^ was one of the strains isolated from an intertidal green algal sample (*Codium fragile*). The isolate was subsequently subjected to the 16S rRNA gene sequence determination. The nearly complete 16S rRNA gene sequence of strain SM1988^T^ (1,502 bp) was obtained through PCR-amplification, which was identical with those obtained from the genomic sequence. The 16S rRNA gene sequence comparison showed that strain SM1988^T^ shared the highest sequence similarity (93.3%) with the type strain of *Pseudoalteromonas mariniglutinosa*, followed by those of *Pseudoalteromonas issachenkonii* (93.2%) and *Pseudoalteromonas tetraodonis* (93.1%), and <93.1% sequence similarity with other known species in the genus *Pseudoalteromonas* and other three genera, i.e., *Algicola* (90.3–92.1%), *Psychrosphaera* (92.2–92.3%) and *Salsuginimonas* (90.6%), in the family *Pseudoalteromonadaceae.* In the neighbor-joining, maximum-likelihood and maximum-parsimony trees based on 16S rRNA gene sequences (Figure 1 and Supplementary Figures S1, S2), strain SM1988^T^ formed a distinct lineage within the *Pseudoalteromonadaceae* clade but sister to the *Pseudoalteromonas* lineage with high bootstrap confidence, clearly indicating that it did not belong to known genera in this family.

The complete genome of strain SM1988^T^ is comprised of a single circular chromosome and is 3973578 bp in length. It totally encodes 3515 protein-coding genes, and the number of RNAs is 94. The DNA G + C content of strain SM1988^T^ calculated from the genome is 39.9 mol%, within the range for the family *Pseudoalteromonadaceae* (Ivanova et al., 2014). The ANI and *is*DDH values between strain SM1988^T^ and type strains of *Pseudoalteromonas mariniglutinosa* KCTC 22327^T^, *Pseudoalteromonas issachenkonii* KMM 3549^T^, *Pseudoalteromonas tetraodonis* GFC^T^, *Algicola sagamiensis* DSM 14643^T^ and *Psychrosphaera saromensis* SA4-48^T^ are below 68.3 and 23.4%, respectively (Supplementary Table S1). All these values are far below the ANI (95%) and DDH (70%) thresholds to discriminate bacterial species. Moreover, the AAI and POCP values between strain SM1988^T^ and the aforementioned type strains are below 55.7 and 47.4%, respectively (Supplementary Table S2). They are also all less than the AAI (70–80%) and POCP (50%) thresholds proposed for genera demarcation (Qin et al., 2014; Skennerton et al., 2015; Wirth and Whitman, 2018; Xu et al., 2019), indicating that strain SM1988^T^ might belong to a new genus distinct from the genera *Pseudoalteromonas*, *Algicola* and *Psychrosphaera*.

### Chemotaxonomic Characteristics

The major cellular fatty acids (>5%) of strain SM1988^T^ were summed feature 8 (C_18:1_ ω7*c*/C_18:1_ ω6*c*) (20.9%), C_17:1_ ω8*c* (19.5%), summed feature 3 (C_16:1_ ω6*c*/C_16:1_ ω7*c*) (17.5%), C_16:0_ (6.8%), and C_17:0_ (5.9%) (Supplementary Table S3). C_18:1_ ω7*c*, C_16:1_ ω6*c*, and C_16:0_ are the common major components in the family *Pseudoalteromonadaceae*. Strain SM1988^T^ contained a pronouncedly higher (20.9% vs. 2.2–4.2%) proportion of summed feature 8 compared with *Pseudoalteromonas mariniglutinosa* DSM 15203^T^, *Pseudoalteromonas haloplanktis* MCCC 1A06496^T^ and *Psychrosphaera haliotis* JCM 16340^T^ (Supplementary Table S3). The polar lipids of strain SM1988^T^ predominantly comprised phosphatidyethanolamine (PE) and phosphatidylglycerol (PG), identical with those of the reference strains shown in Supplementary Figure S3. The sole respiratory quinone of strain SM1988^T^ was ubiquinone-8 (Q-8), in agreement with known species in the family *Pseudoalteromonadaceae* (Ivanova et al., 2014). Overall, chemotaxonomic characteristics of SM1988^T^ support its affiliation to the family *Pseudoalteromonadaceae*.

### Morphological, Physiological, and Biochemical Characteristics

**FIGURE 2:**
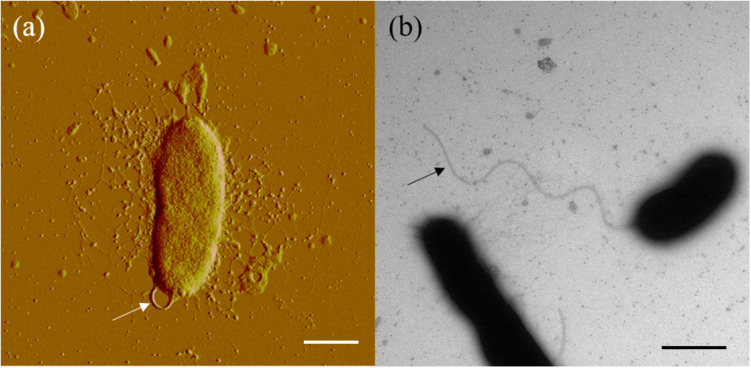
Cell morphological observation of strain SM1988^T^ by AFM **(a)** and TEM **(b)**. Cells for observation were grown in TYS broth medium at 25°C for 10 h. Arrows indicate the positions of flagellum. Bar, 1 μm.

Cells of strain SM1988^T^ were Gram-negative, single polarly flagellated rods (Figure 2). They were oxidase- and catalase-positive and capable of nitrate reduction to nitrite. Colonies on TYS agar incubated at 25°C for 24 h were whitish, circle, smooth, and sticky, but turned slightly yellowish, convex after 48 h of incubation. Strain SM1988^T^ grew at 10–37°C (optimum 25–30°C), pH 6–10 (optimum 8–9), and with 0.5–7.0% (w/v) NaCl (optimum 2.5–3.0%), and showed no growth under anaerobic condition. The optimal temperature, pH, and NaCl concentration for growth of the strain were close to *in situ* seawater temperature, pH, and salinity (21.2°C, pH 8.0 and 3.2%). It formed a large number of flocs after 12 h of incubation in TYS broth at 25°C (Supplementary Figure S4). The strain exhibited a strong ability to hydrolyze casein, gelatin, and Tweens 20, 40, 60, and 80 on TYS agar containing these polymers. It was able to weakly assimilate glucose, arabinose, mannose, mannitol, maltose, *N*-acetyl-glucosamine, gluconate, adipate, malate and citrate. Strain SM1988^T^ was susceptible to erythromycin, chloramphenicol, ampicillin, polymyxin B, colistin sulfate, and oleandomycin. Other phenotypic characteristics were shown in the species description and in Table 1.

**TABLE 1:** Differential characteristics of the genus *Flocculibacter* and known genera in the family *Pseudoalteromonadaceae*.

Characteristic	1	2	3	4	5
Cell shape	Rod	Rod	Rod or coccoid	Rod	Rod
Flagellum	Single, polar	Polar, peritrichous or none	Single, polar	Single, polar	Single, polar
Range for growth					
Temperature (°C)	10–37	4–45	4–37	15–35	20–40
NaCl (%, w/v)	0.5–7.0	0–15.0	0.5–12.0*	0–6.0	0–3.0
Metabolism	Aerobic	Mostly aerobic	Aerobic	Aerobic	Aerobic
Major fatty acids	SF8, C_17:1_ω8*c*, SF3, C_16:0_	C_16:0_, SF3, SF8	C_16:0_, C_17:1_ω8*c*, SF3, SF8	C_16:0_, C_16:1_ω9*c*, SF3, SF8	C_16:0_, SF3, C_17:1_ω8*c*
DNA G + C content (mol%)	39.9	36.8–54.9	38.3–49.5	42–46	51.0
Isolation sources	Algae	Seawater, sediment, marine organisms	Seawater, marine animals, brackish water	Algae, seawater	Brackish river

SF3, summed feature 3 consisted of C_16:1_ω6c/C_16:1_ω7c; SF8, summed feature 8 consisted of C_18:1_ω6c/C_18:1_ω7c. *, sea salt. Genera: (1) Flocculibacter (strain SM1988^T^, this study); (2) Pseudoalteromonas (Ivanova et al., 2014; Park et al., 2016; Yan et al., 2016; Navarro-Torre et al., 2020); (3) Psychrosphaera (Park et al., 2010; Lee et al., 2014; Pheng et al., 2017), (4) Algicola (Sawabe et al., 1998; Kobayashi et al., 2003); (5) Salsuginimonas (Sheu et al., 2017).

### Production and Enzymatic Properties of the Extracellular Proteases

**FIGURE 3:**
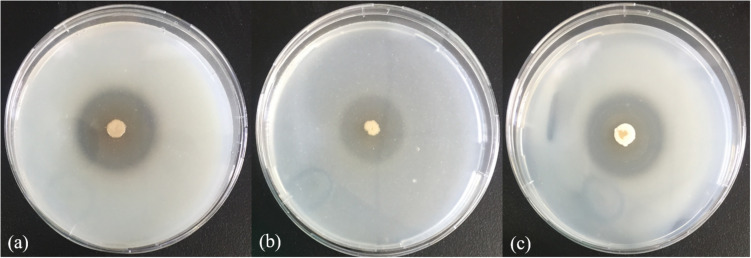
Hydrolysis zones of strain SM1988^T^ on TYS agar containing skim milk (1%, w/v) **(a)**, casein (2%, w/v) **(b)**, and gelatin (2%, w/v) **(c)**. TYS agar plates were incubated at 25°C for 4 days.

**FIGURE 4:**
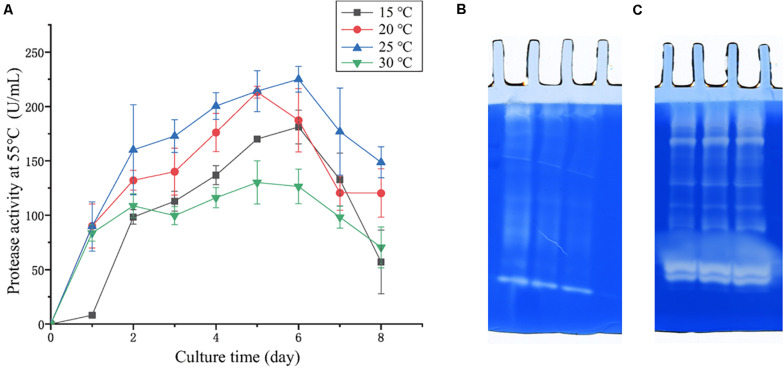
**(A)** Protease production of strain SM1988^T^ cultured at different temperatures in TYS broth medium for 8 days. The extracellular protease activities of the cultures were measured at 55°C in 50 mM Tris-HCl (pH 8.0) with bovine bone collagen as substrate. The graphs show data from triplicate experiments (mean ± SD). **(B)** Casein zymography and **(C)** gelatin zymography of the extracellular proteases produced by strain SM1988^T^ cultured in the TYS broth medium at 25°C for 6 days. The Gelatin and casein zymography were performed according to the method of Heussen and Dowdle (1980). The concentration of gelatin or casein in the gel was 1% (w/v), three lanes in **(B,C)** represent triplicate experiments for casein and gelatin zymography, respectively.

**FIGURE 5:**
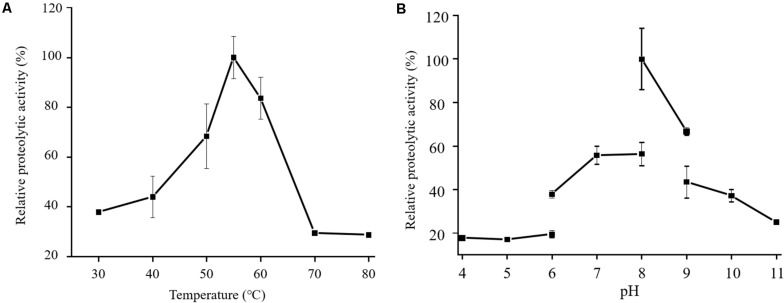
Effects of temperature **(A)** and pH **(B)** on the activity of the proteases secreted by strain SM1988^T^. The culture supernatant of strain SM1988^T^ cultured in the TYS broth medium at 25°C for 6 days was properly diluted 10–100 times in the protease activity assays. The graphs show data from triplicate experiments (mean ± SD). In **(A)**, the protease activities were measured in 50 mM Tris-HCl (pH 8.0) with bovine bone collagen as substrate, and the protease activity at 55°C was taken as 100%. In **(B)**, the protease activities were measured at 55°C with bovine bone collagen as substrate, and the protease activity in 50 mM Tris-HCl (pH 8.0) was taken as 100%.

**FIGURE 6:**
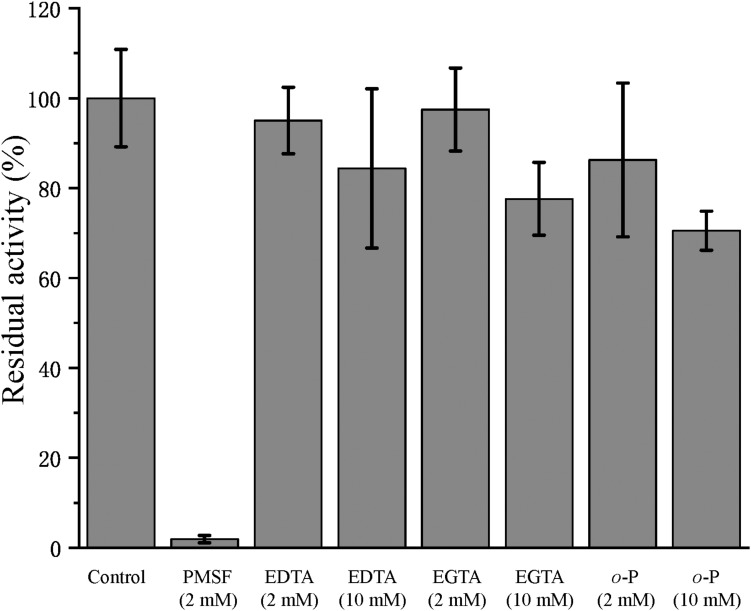
Effects of protease inhibitors on the activity of the collagenase secreted by strain SM1988^T^. The culture supernatant of strain SM1988^T^ cultured in the TYS broth medium at 25°C for 6 days was diluted 100 times with 50 mM Tris-HCl (pH 8.0), and then incubated at 4°C for 1 h with 2 or 10 mM of the inhibitor phenylmethylsulfonyl fluoride (PMSF), ethylene-diamine tetraacetic acid (EDTA), ethylene glycol tetraacetic acid (EGTA), or *o*-phenanthroline (*o*-P). After incubation, the residue protease activity toward bovine bone collagen was measured at 55°C, pH 8.0. The protease activity of the culture supernatant without any inhibitor was taken as 100%.

In the physiological analysis, strain SM1988^T^ was found to show significant hydrolysis on skim milk, casein, and gelatin on agar plates (Figure 3), suggesting that this strain may have a high extracellular protease production. Therefore, we further investigated the production and enzymatic properties of its extracellular protease. In the TYS broth medium, strain SM1988^T^ had the highest protease production at 25°C, which reached 384.14 U/mg (236 U/ml) with insoluble bovine bone collagen as the substrate (Figure 4A and Table 2). The optimal temperature and pH of the extracellular protease toward bovine bone collagen were 55°C and pH 8, respectively (Figure 5). The optimal temperature for the protease activity was the combined effect of many factors, including protease stability, substrate stability (especially for collagen), and the temperature dependence of the reaction etc. As shown in Figure 5B, at pHs 6, 8, and 9, the protease activity showed obvious difference in different buffers, indicating that some buffer components had strong impact on the protease activity. Substrate specificity analysis showed that the extracellular protease could hydrolyze bovine bone collagen fiber, bovine tendon collagen fiber, gelatin, casein, and azocoll at 30 and 55°C, and colfish collagen at 15 and 55°C, but had little activity to elastinorcein (Table 2). This result indicates that strain SM1988^T^ secretes collagenase, and may play a role in collagen degradation in its habitat. Consistently, zymography analysis indicated that proteases with caseinolytic or gelatinolytic activity secreted by strain SM1988^T^ may be diverse, especially those with gelatinolytic activity (Figures 4B,C). The extracellular protease activity of strain SM1988^T^ was almost completely inhibited by the serine protease inhibitor PMSF, whereas metalloprotease inhibitor, EDTA, EGTA, or *o*-P at 10 mM only had a little inhibitory effect (Figure 6), indicating that most of the proteases secreted by strain SM1988^T^ were likely serine proteases.

**TABLE 2:** The enzymatic activities of the extracellular protease of strain SM1988^T^ toward different substrates^$^.

Substrate	Activity (U/mg, 55°C)	Activity (U/mg, 30 or 15°C)
Bovine bone collagen fiber	384.14 ± 7.97	149.59 ± 3.33(30°*C*)
Bovine tendon collagen fiber	241.87 ± 9.08	98.77 ± 2.40(30°*C*)
Codfish collagen	191.36 ± 14.07	163.32 ± 15.78(15°*C*)
Gelatin	767.76 ± 22.66	493.84 ± 20.79(30°*C*)
Casein	147.87 ± 0.69	31.75 ± 3.95(30°*C*)
Azocoll	7.64 ± 2.03	2.38 ± 0.42(30°*C*)
Elastinorcein	0.23 ± 0.02	– (30°C)

*^$^The culture supernatant of strain SM1988^T^ cultured in the TYS broth medium at 25°C for 6 days was 100 times diluted with 50 mM Tris-HCl (pH 8.0), and then the protease activities of the diluted supernatant toward the substrates were measured at the indicated temperatures.*

### Prediction of the Extracellular Proteases Through Genome and Secretome Analyses

The genome of strain SM1988^T^ contains 140 putative proteases (Supplementary Figure S5 and Supplementary Table S4). Among them, 67 proteases are predicted by the SignalP server to have a signal peptide, which are likely extracellular proteases. These predicted extracellular proteases are distributed into 29 families, including 2 families of cysteine peptidases (2/67), 16 families of metallopeptidases (28/67), 10 families of serine peptidases (34/67), and 1 family of peptidases of unknown catalytic type (3/67) (Supplementary Figure S5). Among them, the protease numbers in families S9 (8), S8 (7), S1 (6), and S12 (6) are ≥6, and those in the other families are ≤3.

**FIGURE 7:**
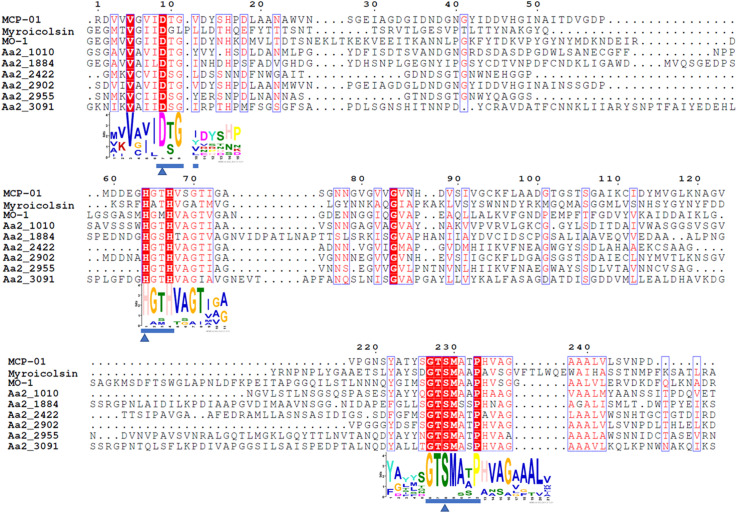
Sequence alignment of the predicted S8 serine proteases (Aa2_1010, Aa2_1884, Aa2_2422, Aa2_2902, Aa2_2955, Aa2_3091) from strain SM1988^T^ and reported S8 collagenases from bacteria using the ClustalW program. Similar amino acid residues are boxed and shown in red, conserved amino acid residues are shown with red background. Amino acid residues constituting catalytic triad of MEROPS S8 family are marked with blue triangles, and motifs containing these residues are marked with blue underlines. MCP-01 (ABD14413), protease from strain MO-1 (BAF30978) and myroicolsin (AEC33275) are reported S8 collagenases produced by strains *Pseudoalteromonas* sp. SM9913 and *Geobacillus* sp. MO-1 and *Myroides profundi* D25, respectively.

**FIGURE 8:**
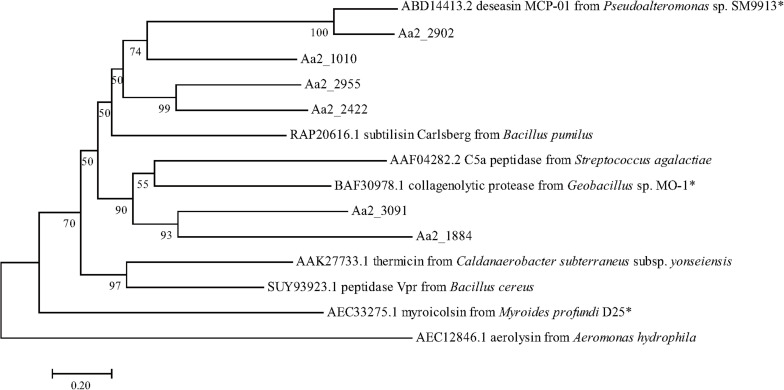
Neighbor-joining (NJ) phylogenetic tree based on amino acids sequences showing the positions of the 6 predicted S8 proteases of strain SM1988^T^ and reported S8 proteases. Bootstrap values (>50%) based on 1,000 replicates were presented at nodes, bar, 0.20. The reported S8 collagenases are indicated by asterisks.

The proteases secreted by strain SM1988^T^ in the TYS broth medium were further identified by secretome analysis. A total of 25 proteases were identified in the secretome (Table 3), including 11 serine proteases, 12 metalloproteases and 2 U69 proteases. Among them, there are 6 serine proteases of the S8 family, which are the most in number (6/25). Some proteases from the S8 family have been reported to be collagenases, including the collagenolytic protease from *Geobacillus* sp. MO-1 (Itoi et al., 2006), deseasin MCP-01 from *Pseudoalteromonas* sp. SM9913 (Chen et al., 2007), and myroicolsin from *Myroides profundi* D25 (Ran et al., 2014). Sequences alignment indicated that all the secreted S8 proteases (Aa2_1010, Aa2_1884, Aa2_2422, Aa2_2902, Aa2_2955, and Aa2_3091) contain similar conserved motifs as these reported S8 collagenases (Figure 7). Phylogenetic analysis showed that Aa2_1010, Aa2_2422, Aa2_2902, and Aa2_2955 are closely related to deseasin MCP-01, and Aa2_1884 and Aa2_3091 are closely related to the collagenolytic protease from *Geobacillus* sp. MO-1 (Figure 8). These data suggest that these extracellular S8 serine proteases are potential collagenases. Therefore, combined with the above inhibitor experiment result (Figure 6), it can be concluded that all or some of the S8 serine proteases secreted by strain SM1988^T^ likely contribute to its extracellular collagenase activity.

**TABLE 3:** Proteases detected in the secretome of strain SM1988^T^.

Family	Protease	Coverage	Peptide number^a^	PSMs^b^	Predicted functions^c^
S8	Aa2_1884	12.07	12	143	Degrading extracellular proteins for microbial nutrition, and some have collagenolytic activity.
	Aa2_2902	15.40	12	59	
	Aa2_3091	10.17	10	15	
	Aa2_2955	11.45	8	14	
	Aa2_1010	6.69	4	9	
	Aa2_2422	9.59	4	5	
S1	Aa2_0587	22.38	9	49	Intestinal digestion and IgA-mediated immune response etc.
	Aa2_0495	2.76	2	2	
S9	Aa2_1209	18.84	12	16	Degrading biologically active peptides.
	Aa2_0579	19.33	10	13	
	Aa2_0055	11.58	8	8	
M14	Aa2_0988	32.60	18	59	Digestion of food, processing of bioactive peptides, and the metabolism of bacterial cell walls.
	Aa2_1984	12.61	10	24	
M61	Aa2_2501	35.70	25	78	Degrading extracellular proteins.
M97	Aa2_0406	24.78	21	40	Endopeptidase activity.
M72	Aa2_1900	23.90	10	26	Endopeptidase activity.
	Aa2_2425	18.33	3	4	
M6	Aa2_0830	9.31	6	12	Involved in protein–protein or protein–carbohydrate interactions.
M35	Aa2_2852	18.13	5	12	Endopeptidase activity.
M2	Aa2_1365	8.39	5	5	Involved in blood homoeostasis and reproduction.
M13	Aa2_2814	7.96	5	5	Having a nutritional role.
M16	Aa2_2635	9.56	4	5	Clearance of small peptides.
M28	Aa2_2022	8.16	4	4	Catalyzing the removal of a wide range of N-terminal amino acid residues from peptides and proteins.
U69	Aa2_0983	43.60	25	220	Involved in adhesion, biofilm formation, aggregation and invasion into host cells.
	Aa2_0187	10.71	10	29	

*^a^FDR for peptide identification was set to 0.01. ^b^Peptide spectrum matches. ^c^Functions were predicted based on the MEROPS website except that of the S8 proteases based on both the MEROPS website and references (Itoi et al., 2006; Zhao et al., 2008; Ran et al., 2014; Zhang et al., 2015).*

## Conclusion

In this study, a *Pseudoalteromonas*-like gammaproteobacterium (strain SM1988^T^) having a strong ability to hydrolyze collagen was isolated from an intertidal green algal sample. Phylogenetic analysis based on the 16S rRNA gene sequences revealed that this bacterium occupied a distinct branch within the clade of the family *Pseudoalteromonadaceae*, which clearly indicates that the strain belongs to a new genus separated from the known genera (*Algicola*, *Pseudoalteromonas*, *Psychrosphaera* and *Salsuginimonas*) in the family. The calculation of two genome-based metrics (AAI and POCP) for genus demarcation between this strain and the type strains selected in known genera of the family *Pseudoalteromonadaceae* further supported its affiliation with a novel genus. Strain SM1988^T^ (the genus *Flocculibacter*) possesses typical chemotaxonomic characteristics in the family *Pseudoalteromonadaceae*, including C_16:0_, C_16:1_ ω7*c*, and C_18:1_ ω7*c* as the major fatty acids, Q-8 as the respiratory quinone, and PE and PG as the major polar lipids. However, strain SM1988^T^ (the genus *Flocculibacter*) could be differentiated from known genera in the family *Pseudoalteromonadaceae* by many characteristics (Table 1). For example, it differs from the genus *Pseudoalteromonas*, the most closely related genus, by its unipolar flagellation, its inability to grow below 10°C and its possessing of C_17:1_ ω8*c* as one of the major fatty acids and relatively low DNA G + C content. In conclusion, based on the results from phylogenetic analysis of the 16S rRNA gene sequences, genome analysis, chemotaxonomic and phenotypic characterization and comparison for strain SM1988^T^, it should be allocated into a new genus in the family *Pseudoalteromonadaceae* as a representative of a novel species, for which the name *Flocculibacter collagenilyticus* gen. nov., sp. nov. is proposed. Furthermore, it was found that strain SM1988^T^ had a high protease production of 384.14 U/mg (236 U/mL). The secreted proteases have high enzymatic activity against both bovine bone collagen and codfish skin collagen. Biochemical tests combined with genome and secretome analyses suggested that the MEROPS S8 serine proteases secreted by SM1988^T^ are potential collagenases, and likely contribute to the extracellular collagenolytic activity. These data suggest that this strain plays a role in marine collagen degradation. This study lays a foundation for further exploring the ecological role of strain SM1988^T^ in collagen degradation and recycling in marine environments and for exploiting its application potential in collagen resource utilization.

### Description of *Flocculibacter* gen. nov.

*Flocculibacter* (Floc.cu.li.bac’ter. N.L. masc. n. *flocculus* a little floc; N.L. masc. n. *bacter* a rod; N.L. masc. n. *Flocculibacter* a rod that produces little flocs).

Cells are Gram-negative, aerobic, oxidase-, and catalase-positive, flagellated rods. The major cellular fatty acids are summed feature 8 (C_18:1_ ω7*c*/C_18:1_ ω6*c*), C_17:1_ ω8*c*, summed feature 3 (C_16:1_ ω7*c*/C_16:1_ ω6*c*), and C_16:0_. The major polar lipids are phosphatidylethanolamine (PE) and phosphatidylglycerol (PG). The predominant respiratory quinone is ubiquinone-8 (Q-8). The genus phylogenetically belongs to the family *Pseudoalteromonadaceae* in the class *Gammaproteobacteria*.

The type species is *Flocculibacter collagenilyticus*.

### Description of *Flocculibacter collagenilyticus* sp. nov.

*Flocculibacter collagenilyticus* (col.la.ge.ni.ly’ti.cus. N.L. neut. n. *collagenum* collagen; N.L. masc. adj. *lyticus* (from Gr. masc. adj. *lytikos*) dissolving; N.L. masc. adj. *collagenilyticus* collagen-dissolving).

The species exhibits the following characteristics in addition to those given in the genus description. Cells are 0.8–1.5 μm long and 0.3–0.5 μm wide when grown in TYS broth at 25°C for 12 h. Cells grows at 10–37°C (optimum, 25–30°C), at pH 6–10 (optimum, pH 8–9), and with 0.5–7.0% (w/v) NaCl (optimum, 2.5–3.0%). Cells are positive for hydrolysis of collagens, casein, gelatin, and Tweens 20, 40, 60, and 80, but negative for hydrolysis of starch. In API 20NE tests, cells are positive for nitrate reduction, aesculin hydrolysis (weakly), gelatin hydrolysis, and assimilation (weakly) of glucose, arabinose, mannose, mannitol, maltose, *N*-acetyl-glucosamine, gluconate, adipate, malate, and citrate but negative for indole production, acid production from glucose, arginine dihydrolase, urease, *β*-galactosidase, and assimilation of caprate and phenylacetate. In the API ZYM tests, alkaline phosphatase, esterase (C4, weak), esterase lipase (C8, weak), leucine arylamidase, valine arylamidase, cystine arylamidase (weak), α-chymotrypsin (weak), acid phosphatase, naphthol-AS-BI-phosphohydrolase are present, but lipase (C14), trypsin, *α*-galactosidase, *β*-galactosidase, *β*-glucuronidase, *α*-glucosidase, *β*-glucosidase, *N*-acetyl-*β*-glucosaminidase, *α*-mannosidase, *β*-fucosidase are absent. Cells are susceptible to erythromycin, chloramphenicol, ampicillin, polymyxin B, colistin sulfate, oleandomycin, carbenicillin, penicillin G, tetracycline, gentamicin, and novobiocin, kanamycin. Except for major cellular fatty acids in the genus description, the following fatty acids are also detected as minor ones (>2% but <6%): C_17:0_, C_10:0_ 3-OH, C_18:0_, C_12:0_ 3-OH, and C_12:0_.

The type strain is SM1988^T^ (= MCCC 1K04279^T^ = KCTC 72761^T^) isolated from a green algae *Codium fragile* sample collected from the intertidal zones of Qingdao City, China. The genomic G + C content of the type strain determined based on the complete genome is 39.9 mol%.

## Data Availability Statement

The datasets presented in this study can be found in online repositories. The names of the repository/repositories and accession number(s) can be found below: https://www.ncbi.nlm.nih.gov/genbank/, MN606291. https://www.ncbi.nlm.nih.gov/genbank/, CP059888. https://www.ebi.ac.uk/pride, PXD023431.

## Author Contributions

X-LC, Y-ZZ, XT, and X-YZ designed and coordinated the study. JL performed all the experiments and drafted the manuscript. J-HC and Z-JT helped in enzymatic characterization. X-YH helped in sample collecting and experiments. Z-ZS, PW, MS, and X-YS helped in data analysis and interpretation. X-LC and X-YZ revised the manuscript. All authors contributed to the article and approved the submitted version.

## Conflict of Interest

The authors declare that the research was conducted in the absence of any commercial or financial relationships that could be construed as a potential conflict of interest.

## Footnotes

1 https://lpsn.dsmz.de

2 https://www.ezbiocloud.net

3 https://github.com/tseemann/barrnap

4 http://merops.sanger.ac.uk

5 http://ggdc.dsmz.de

6 http://jspecies.ribohost.com/jspeciesws

7 http://enve-omics.ce.gatech.edu/aai/

8 http://espript.ibcp.fr/ESPript/cgi-bin/ESPript.cgi
