# Supplementary material for: Taxonomic and Enzymatic Characterization of Flocculibacter collagenilyticus gen. nov., sp. nov., a Novel Gammaproteobacterium With High Collagenase Production
Source: Front Microbiol. 2021 Mar 9;12:621161. doi: 10.3389/fmicb.2021.621161 (PMC8005334; doi:10.3389/fmicb.2021.621161)
Supplement: Supplementary file 1 [file Data_Sheet_1.docx]

Supplementary Figures and Tables for

Taxonomic and enzymatic characterization of *Flocculibacter collagenilyticus* gen. nov., sp. nov., a novel gammaproteobacterium with high collagenase production

Jian Li^1,2^, Jun-Hui Cheng^2^, Zhao-Jie Teng^2^, Zhong-Zhi Sun^2^, Xiao-Yan He^2^, Peng Wang^3,4^, Mei Shi^2^, Xiao-Yan Song^2^, Xiu-Lan Chen^2^, Yu-Zhong Zhang^2,3,4^, Xinmin Tian^1^*, Xi-Ying Zhang^2^*

^1^ College of Life Science and Technology, Xinjiang University, Urumqi, China

^2^State Key Laboratory of Microbial Technology, Institute of Marine Science and Technology, Marine Biotechnology Research Center, Shandong University, Qingdao, China

^3^Laboratory for Marine Biology and Biotechnology, Pilot National Laboratory for Marine Science and Technology, Qingdao, China

^4^College of Marine Life Sciences, and Frontiers Science Center for Deep Ocean Multispheres and Earth System, Ocean University of China, Qingdao, China


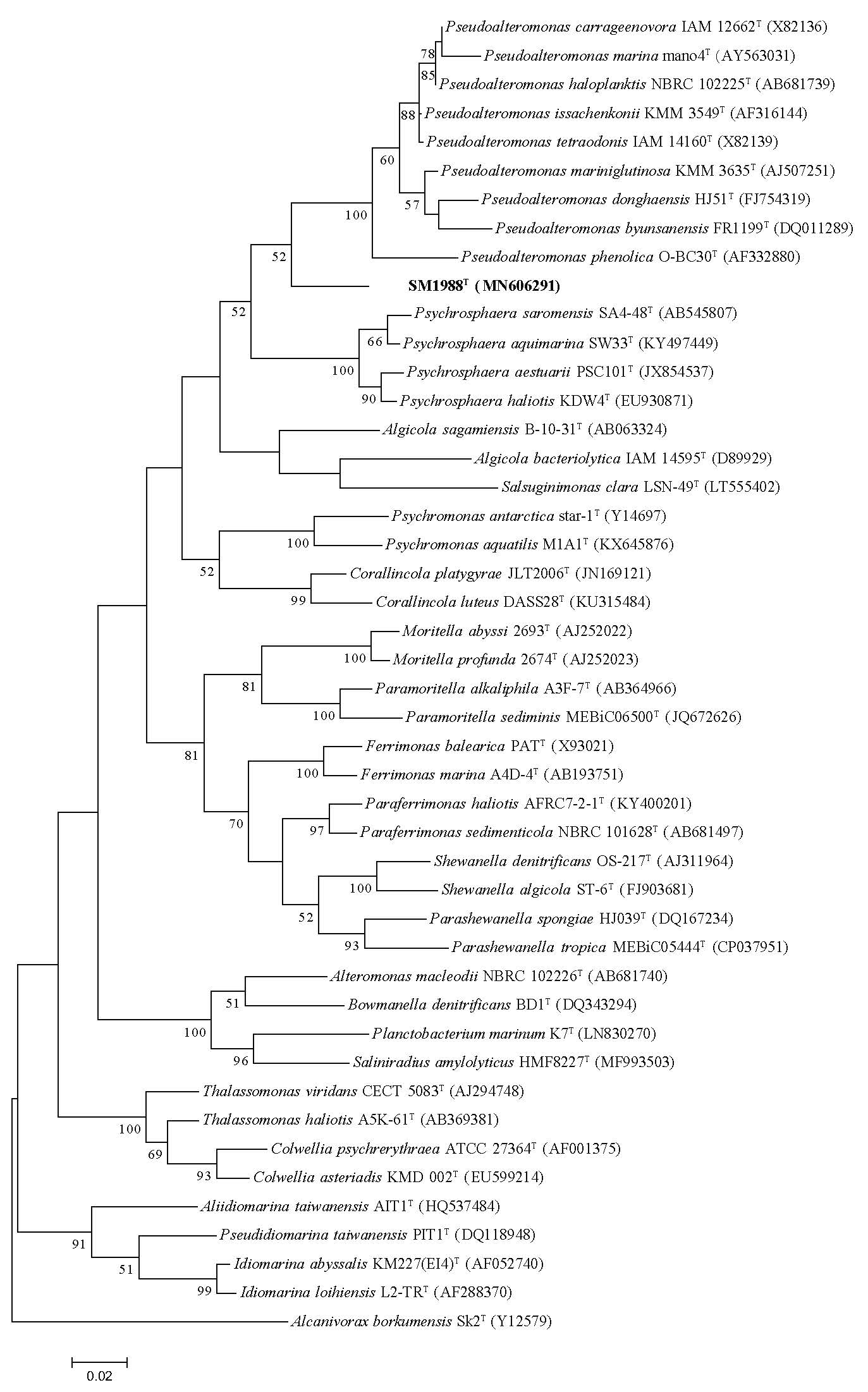


**Supplementary Figure S1**. Maximum-likelihood (ML) phylogenetic tree based on 16S rRNA gene sequences showing the positions of strain SM1988^T^ (in bold) and selected known species in the family *Pseudoalteromonadaceae* and other closely related families in the class *Gammaproteobacteria*. Bootstrap values (>50%) based on 1000 replicates are presented at nodes. *Alcanivorax borkumensis* Sk2^T^ was selected for outgroup. Bar, 0.02 substitutions per nucleotide position.


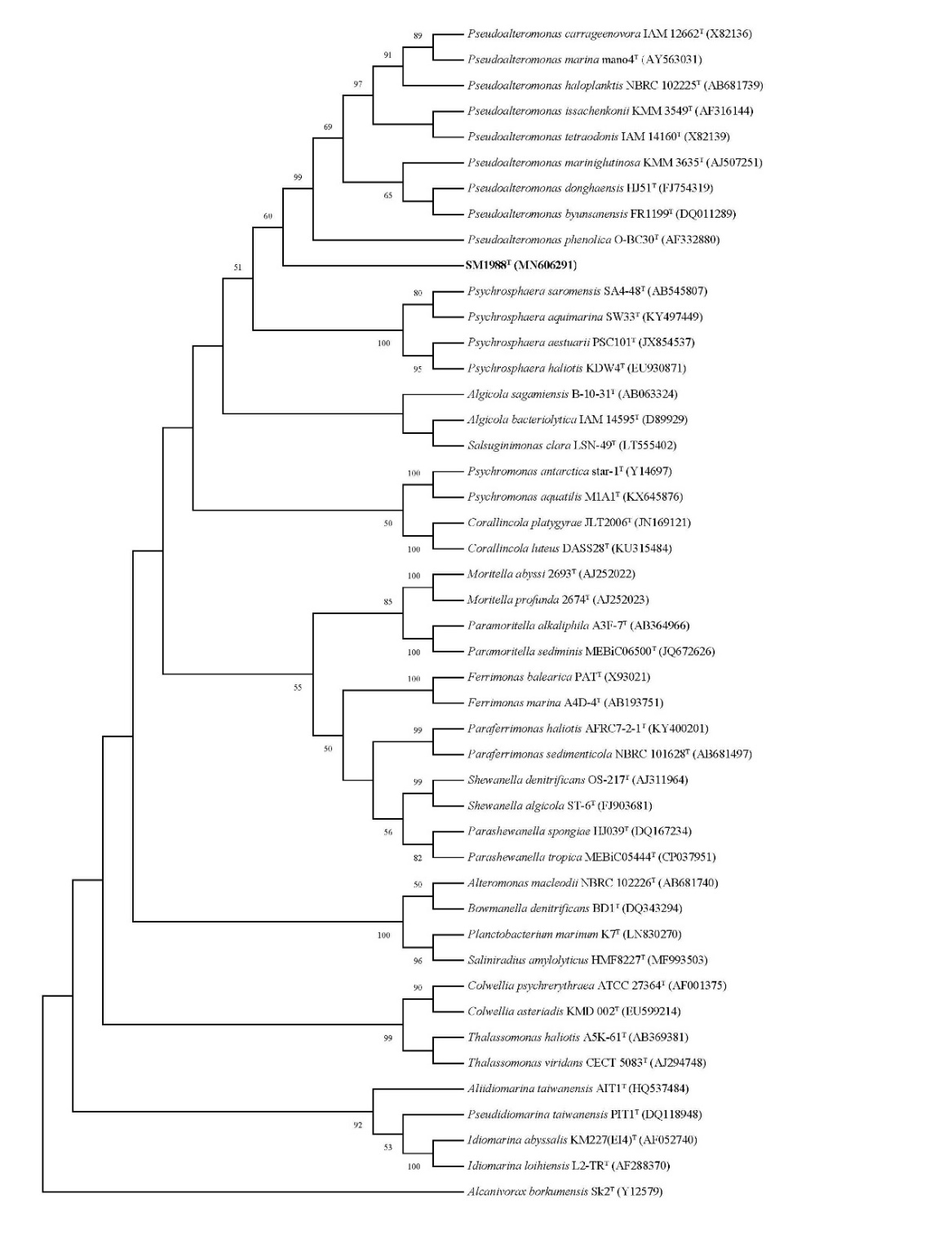


**Supplementary Figure S2**. Maximum-parsimony (MP) phylogenetic tree based on 16S rRNA gene sequences showing the positions of strain SM1988^T^ (in bold) and selected known species in the family *Pseudoalteromonadaceae* and other closely related families in the class *Gammaproteobacteria*. Bootstrap values (>50%) based on 1000 replicates are presented at nodes. *Alcanivorax borkumensis* Sk2^T^ was selected for outgroup.


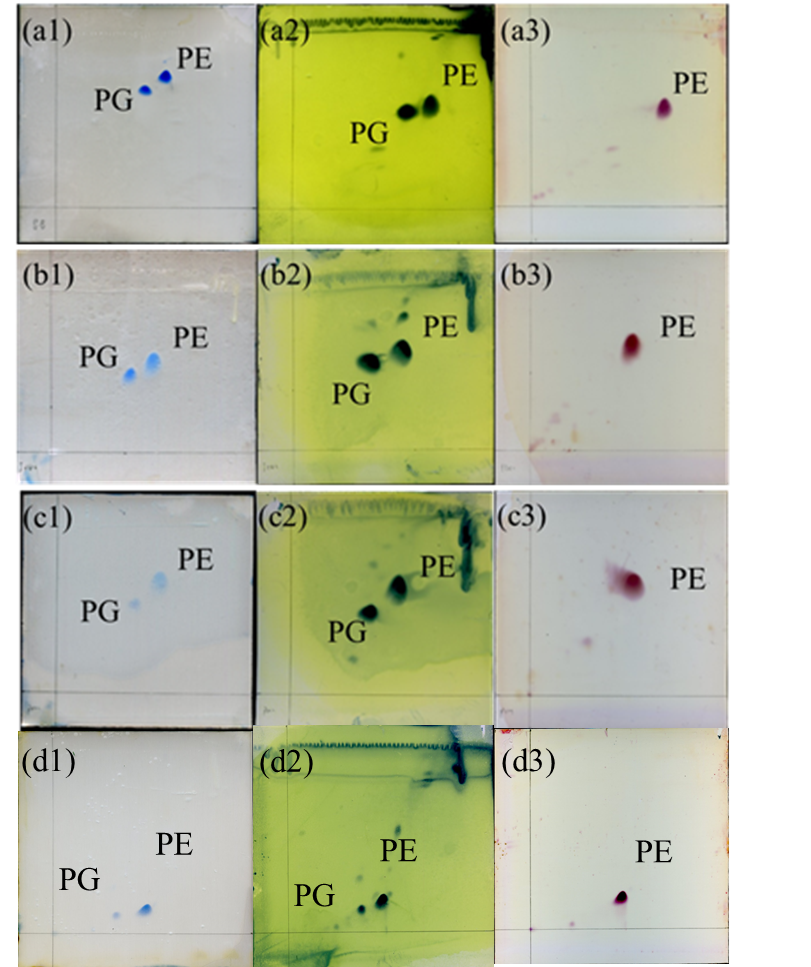


**Supplementary Figure S3**. Two-dimensional TLCs of polar lipids of *Flocculibacter collagenilyticus* SM1988^T^ (a), *Pseudoalteromonas mariniglutinosa* DSM 15203^T^ (b), *Psychrosphaera haliotis* JCM 16340^T^ (c), and *Pseudoalteromonas haloplanktis* MCCC 1A06496^T^ (d),which respectively stained by molybdenum blue (a1, b1, c1 and d1), ethanolic molybdophosphoric acid (a2, b2, c2 and d2) and ninhydrin reagent (a3, b3, c3 and d3). PE, phosphatidylethanolamine; PG, phosphatidylglycerol.


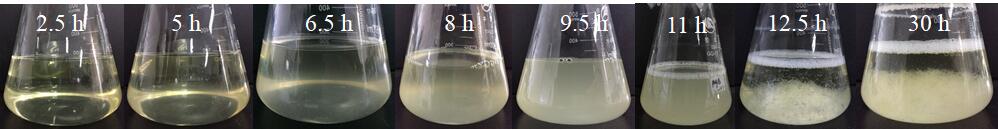


**Supplementary** **Figure S4**. The flocs formation of SM1988^T^ during the culturing course (2.5-30 h).


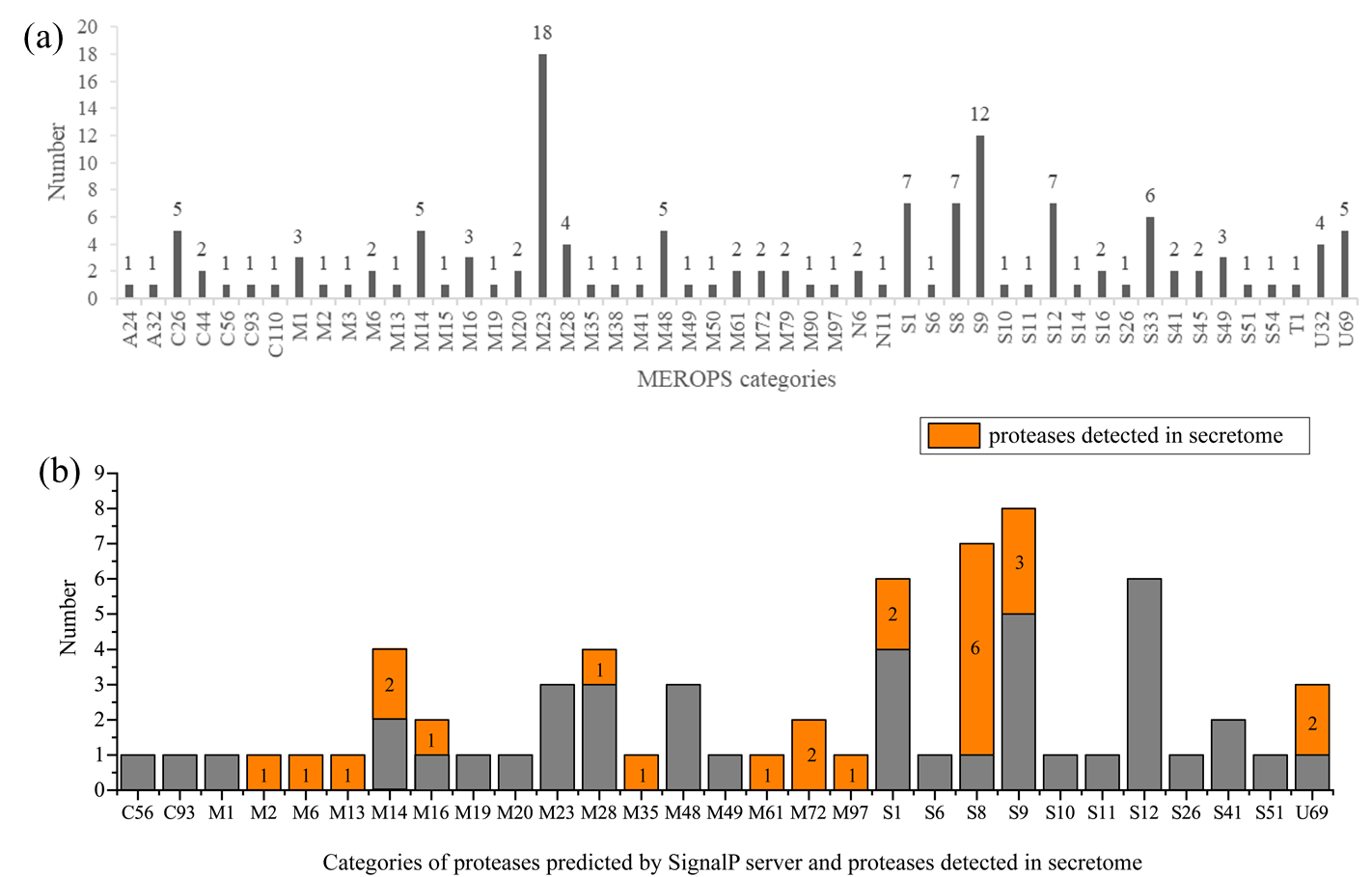


**Supplementary Figure S5**. The categories and numbers of the all proteases of strain SM1988^T^ predicted by MEROPS (a) and of the proteases predicted by SignalP server and detected in secretome (b). The proteases detected in secretome are in orange, and their counts are marked.

**Supplementary Table S1.** Average nucleotide identity (ANI) and Genome-to-Genome-Distance (GGDC) comparisons between strain SM1988^T^ and type strains of closely related species in the family *Pseudoalteromonadaceae*.

| Strains | ANI value (%) | | GGDC value (%) | Accession numbers |
| --- | --- | --- | --- | --- |
|  | ANIb | ANIm |  |  |
| *Algicola sagamiensis* DSM 14643^T^ | 67.4 | 84.4 | 22.1 | NZ_ARFX00000000 |
| *Psychrosphaera saromensis* SA4-48^T^ | 68.3 | 84.4 | 23.4 | NZ_MSCH00000000 |
| *Pseudoalteromonas mariniglutinosa* KCTC 22327^T^ | 68.2 | 84.3 | 21.7 | NZ_BDDU01000000 |
| *Pseudoalteromonas issachenkonii* KMM 3549^T^ | 68.2 | 83.9 | 21.9 | NZ_CP011030, NZ_CP011031 |
| *Pseudoalteromonas tetraodonis* GFC^T^ | 68.3 | 84.2 | 22.4 | NZ_CP011041,  NZ_CP011042 |

**Supplementary Table S2**. The Average Amino Acid Identity (AAI) and the Percentage of Conserved Proteins (POCP) values between strain SM1988^T^ and type strains of closely related species in the family *Pseudoalteromonadaceae*.

| Strains | AAI value (%) | POCP value (%) | Accession numbers |
| --- | --- | --- | --- |
| *Algicola sagamiensis* DSM 14643^T^ | 54.9 | 41.8 | NZ_ARFX00000000 |
| *Psychrosphaera saromensis* SA4-48^T^ | 55.1 | 46.8 | NZ_MSCH00000000 |
| *Pseudoalteromonas mariniglutinosa* KCTC 22327^T^ | 55.5 | 45.2 | NZ_BDDU01000000 |
| *Pseudoalteromonas issachenkonii* KMM 3549^T^ | 55.7 | 47.4 | NZ_CP011030, NZ_CP011031 |
| *Pseudoalteromonas tetraodonis* GFC^T^ | 55.5 | 47.2 | NZ_CP011041,  NZ_CP011042 |

**Supplementary Table S3**. Fatty acid compositions (%) of strain SM1988^T^ (1) and type strains of *Pseudoalteromonas mariniglutinosa* DSM 15203^T^ (2), *Psychrosphaera haliotis* JCM 16340^T^ (3), and *Pseudoalteromonas haloplanktis* MCCC 1A06496^T^ (4). ^$^

| Fatty acids | 1 | 2 | 3 | 4 |
| --- | --- | --- | --- | --- |
| Straight-chain fatty acids |  |  |  |  |
| C_9:0_ | 0.8 | - | 1.4 | - |
| C_10:0_ | 1.1 | - | - | - |
| C_12:0_ | 2.4 | 1.3 | 0.7 | 1.9 |
| C_13:0_ | 1.7 | 1.1 | 1.6 | 1.5 |
| C_14:0_ | 1.1 | 1.5 | 0.8 | 2.2 |
| C_16:0_ | 6.8 | **13.5** | **10.6** | **10.8** |
| C_17:0_ | 5.9 | 6.5 | 9.2 | 3.1 |
| C_18:0_ | 3.0 | 0.8 | 1.0 | 0.7 |
| Unsaturated fatty acids |  |  |  |  |
| C_15:1_ *ω*6*c* | 0.3 | 0.7 | 0.3 | 1.5 |
| C_15:1_ *ω*8*c* | 1.6 | 4.9 | **13.1** | 6.7 |
| C_17:1_ *ω*6*c* | - | 1.1 | - | 0.8 |
| C_17:1_ *ω*8*c* | **19.5** | **13.6** | **22.5** | **11.6** |
| C_18:1_ *ω*7*c* 11-methyl | - | - | 3.7 | - |
| Branched fatty acids |  |  |  |  |
| iso-C_14:0_ | - | 1.1 | 0.6 | 1.0 |
| iso-C_15:0_ | - | 0.2 | 0.1 | 1.6 |
| iso-C_16:0_ | 1.3 | **11.0** | 5.3 | 5.6 |
| iso-C_17:0_ | 0.2 | 0.9 | 0.3 | 1.9 |
| iso-C_18:0_ | 0.9 | 2.2 | 1.2 | 0.5 |
| Hydroxy fatty acids |  |  |  |  |
| C_10:0_ 3-OH | 3.4 | 0.2 | 2.0 | 0.5 |
| C_11:0_ 3-OH | 1.4 | 0.8 | 2.7 | 1.3 |
| C_12:0_ 3-OH | 2.6 | 2.4 | 0.2 | 3.7 |
| iso-C_11:0_ 3-OH | 0.5 | 0.2 | 0.4 | 1.0 |
| iso-C_12:0_ 3-OH | 0.1 | 4.5 | 1.0 | 2.3 |
| Summed feature^*^ |  |  |  |  |
| 1 | 0.88 |  | 0.03 | 1.0 |
| 3 | **17.5** | **22.8** | **12.5** | **29.4** |
| 8 | **20.9** | 4.2 | 3.5 | 2.2 |

^$^ The strains were grown on TYS agar at 25°C for 3 d. All data listed in the table are from this study. “-”, not detected; fatty acids present at > 10% are indicated in bold.

* Summed feature 3 consisted of C_16:1_ *ω*7*c*/C_16:1_ *ω*6*c*, and summed feature 8 consisted of C_18:1_ *ω*7*c*/C_18:1_ *ω*6*c*.

**Supplementary Table S4**. A list of predicted proteases of strain SM1988^#^

| Predicted protease | MEROPS analysis | | | | Predicted signal peptide | Secretome analysis | |
| --- | --- | --- | --- | --- | --- | --- | --- |
|  | Closest homolog | Annotation | Identity (%) | E-value |  | Detected | PSMs |
| Aa2_0049 | MER0124066 | subfamily S8B unassigned peptidases | 32.075 | 1.71E-07 | Y | Y | 6 |
| Aa2_0055 | MER0326619 | subfamily S9C trilobed protease | 90.347 | 1.39E-174 | Y | Y | 8 |
| Aa2_0068 | MER0841469 | family M20D unassigned peptidases | 73.096 | 0 | Y | N | - |
| Aa2_0098 | MER0147154 | subfamily M16B unassigned peptidases | 33.107 | 5.17E-101 | Y | N | - |
| Aa2_0115 | MER0243846 | subfamily M28F unassigned peptidases | 46.97 | 2.1E-81 | Y | N | - |
| Aa2_0146 | MER0064803 | family M19 unassigned peptidases | 73.656 | 0 | Y | N | - |
| Aa2_0187 | MER0283261 | family U69 unassigned peptidases | 33.333 | 2.77E-13 | Y | Y | 29 |
| Aa2_0210 | MER0057864 | family S33 unassigned peptidases | 42.515 | 5.69E-84 | N | N | - |
| Aa2_0265 | MER0083657 | subfamily M28F unassigned peptidases | 61.29 | 4.27E-136 | Y | N | - |
| Aa2_0266 | MER0246330 | subfamily S1C unassigned peptidases | 67.55 | 6.26E-68 | Y | N | - |
| Aa2_0291 | MER0501945 | family U32 unassigned peptidases | 53.03 | 6.09E-13 | N | N | - |
| Aa2_0299 | MER0052732 | family M1 unassigned peptidases | 87.556 | 8.03E-147 | Y | N | - |
| Aa2_0332 | MER0275241 | family U69 unassigned peptidases | 41.297 | 1.7E-38 | N | N | - |
| Aa2_0334 | MER0469311 | family S6 unassigned peptidases | 41.096 | 5.84E-09 | Y | N | - |
| Aa2_0406 | MER0487223 | family M97 unassigned peptidases | 31.797 | 7.61E-28 | Y | Y | 40 |
| Aa2_0430 | MER0501642 | subfamily M23B unassigned peptidases | 33.898 | 2.55E-16 | N | N | - |
| Aa2_0438 | MER0080478 | subfamily M14B unassigned peptidases | 64.669 | 1.12E-144 | Y | N | - |
| Aa2_0440 | MER0092587 | family S33 unassigned peptidases | 48.718 | 2.75E-72 | N | N | - |
| Aa2_0451 | MER0195833 | family N11 unassigned peptide lyase | 36.25 | 5.3E-10 | N | N | - |
| Aa2_0457 | MER0501642 | subfamily M23B unassigned peptidases | 48.864 | 2.31E-17 | N | N | - |
| Aa2_0495 | MER0503884 | subfamily S1A unassigned peptidases | 51.899 | 2.17E-74 | Y | Y | 2 |
| Aa2_0517 | MER0089738 | subfamily M23B unassigned peptidases | 63.158 | 1.6E-78 | N | N | - |
| Aa2_0522 | MER0689043 | subfamily A24A unassigned peptidases | 66.818 | 2.12E-106 | N | N | - |
| Aa2_0527 | MER0241943 | family A32 unassigned peptidases | 50.365 | 4.06E-38 | N | N | - |
| Aa2_0579 | MER0192182 | subfamily S9A unassigned peptidases | 80.986 | 1.97E-171 | Y | Y | 13 |
| Aa2_0587 | MER0503884 | subfamily S1A unassigned peptidases | 61.373 | 3.59E-98 | Y | Y | 49 |
| Aa2_0588 | MER0501642 | subfamily M23B unassigned peptidases | 34.921 | 6.8E-18 | N | N | - |
| Aa2_0606 | MER0250065 | subfamily S9B unassigned peptidases | 65.679 | 0 | Y | N | - |
| Aa2_0613 | MER0076303 | family M49 unassigned peptidases | 79.874 | 4.41E-88 | Y | N | - |
| Aa2_0627 | MER0501642 | subfamily M23B unassigned peptidases | 32.995 | 1.11E-17 | Y | N | - |
| Aa2_0668 | MER0501642 | subfamily M23B unassigned peptidases | 47.5 | 1.18E-15 | N | N | - |
| Aa2_0671 | MER0060859 | family T1B HslV component of HslUV peptidase | 86.628 | 2.99E-109 | N | N | - |
| Aa2_0682 | MER0248680 | family C44 unassigned peptidases | 61.569 | 1.57E-109 | N | N | - |
| Aa2_0830 | MER0042035 | family M6 unassigned peptidases | 40.702 | 5.3E-56 | Y | Y | 12 |
| Aa2_0841 | MER0504954 | subfamily M23B Mername AA292 peptidase | 56.522 | 3.51E-133 | Y | N | - |
| Aa2_0861 | MER0889419 | subfamily M48C GSU1437 putative peptidase | 71.121 | 1.77E-122 | Y | N | - |
| Aa2_0874 | MER0935533 | family M79 unassigned peptidases | 66.892 | 3.75E-61 | N | N | - |
| Aa2_0883 | MER0118163 | family S12 unassigned peptidases | 66.462 | 1.65E-161 | Y | N | - |
| Aa2_0903 | MER0014731 | family C56 unassigned peptidases | 54.217 | 2.85E-57 | Y | N | - |
| Aa2_0915 | MER0239797 | family M38 unassigned peptidases | 50 | 1.01E-128 | N | N | - |
| Aa2_0983 | MER0254557 | family U69 unassigned peptidases | 39.815 | 1.75E-10 | Y | Y | 220 |
| Aa2_0987 | MER0939438 | family M79 unassigned peptidases | 32.558 | 5.1E-14 | N | N | - |
| Aa2_0988 | MER1152098 | subfamily M14A unassigned peptidases | 72.897 | 1.94E-171 | Y | Y | 59 |
| Aa2_0992 | MER0042396 | subfamily M48C unassigned peptidases | 35.827 | 4.72E-46 | N | N | - |
| Aa2_0994 | MER0145363 | family S9 unassigned peptidases | 56.818 | 1.27E-87 | Y | N | - |
| Aa2_1010 | MER0388172 | subfamily S8A Apr peptidase | 70.552 | 3.13E-163 | Y | Y | 9 |
| Aa2_1026 | MER0249591 | subfamily M48C Oma1 peptidase | 65.086 | 9.15E-109 | Y | N | - |
| Aa2_1034 | MER0501642 | subfamily M23B unassigned peptidases | 32.663 | 1.2E-18 | N | N | - |
| Aa2_1066 | MER0405435 | family N6 FlhB protein | 67.647 | 9.78E-177 | N | N | - |
| Aa2_1140 | MER0501642 | subfamily M23B unassigned peptidases | 40 | 3.17E-15 | N | N | - |
| Aa2_1157 | MER0493483 | subfamily S49B unassigned peptidases | 51.94 | 9.12E-120 | N | N | - |
| Aa2_1212 | MER0838207 | subfamily M15B unassigned peptidases | 52.795 | 9.96E-55 | N | N | - |
| Aa2_1213 | MER0248202 | subfamily M20A DapE peptidase | 73.315 | 0 | N | N | - |
| Aa2_1254 | MER0159794 | family S12 unassigned peptidases | 68.06 | 4.55E-174 | Y | N | - |
| Aa2_1294 | MER0083865 | family S12 unassigned peptidases | 75 | 4.36E-172 | Y | N | - |
| Aa2_1306 | MER0357921 | family S14 peptidase Clp | 89.796 | 1.05E-132 | N | N | - |
| Aa2_1308 | MER0297133 | family S16 Lon A peptidase | 82.186 | 2.74E-142 | N | N | - |
| Aa2_1342 | MER0995275 | subfamily S9A unassigned peptidases | 60.156 | 1E-114 | Y | N | - |
| Aa2_1365 | MER0816779 | family M2 unassigned peptidases | 78.772 | 0 | Y | Y | 5 |
| Aa2_1389 | MER0501642 | subfamily M23B unassigned peptidases | 30.682 | 2.22E-13 | N | N | - |
| Aa2_1430 | MER1062362 | subfamily S41A unassigned peptidases | 56.962 | 1.29E-119 | Y | N | - |
| Aa2_1433 | MER0273788 | family M1 unassigned peptidases | 68.9 | 0 | N | N | - |
| Aa2_1472 | MER0501945 | family U32 unassigned peptidases | 46.377 | 3.55E-06 | N | N | - |
| Aa2_1482 | MER0066012 | subfamily S49B unassigned peptidases | 63.068 | 4.01E-71 | N | N | - |
| Aa2_1501 | MER0904425 | family M61 unassigned peptidases | 52.837 | 0 | N | Y | 6 |
| Aa2_1511 | MER0309080 | subfamily M14A unassigned peptidases | 67.626 | 1.07E-141 | N | N | - |
| Aa2_1521 | MER0320677 | subfamily S49B unassigned peptidases | 54.655 | 0 | N | N | - |
| Aa2_1524 | MER0075049 | family M6 unassigned peptidases | 79.026 | 8.45E-159 | N | Y | 11 |
| Aa2_1532 | MER0501642 | subfamily M23B unassigned peptidases | 31.977 | 2.85E-20 | N | N | - |
| Aa2_1612 | MER0244753 | family N6 unassigned peptide lyases | 40.762 | 1.63E-79 | N | N | - |
| Aa2_1641 | MER0132866 | family S33 unassigned peptidases | 50.192 | 1.11E-87 | N | Y | 4 |
| Aa2_1734 | MER0473019 | family S12 unassigned peptidases | 32.68 | 1.14E-49 | Y | N | - |
| Aa2_1742 | MER0627270 | family C110 unassigned peptidases | 66.667 | 7.41E-28 | N | N | - |
| Aa2_1743 | MER0296109 | subfamily M48B HtpX peptidase | 75 | 2.26E-132 | N | N | - |
| Aa2_1865 | MER0441860 | family C26 unassigned peptidases | 36.567 | 1.39E-21 | N | N | - |
| Aa2_1883 | MER0097251 | family S54 unassigned peptidases | 48.193 | 4.1E-48 | N | N | - |
| Aa2_1884 | MER0055868 | subfamily S8A unassigned peptidases | 44.301 | 1.92E-123 | Y | Y | 143 |
| Aa2_1900 | MER0386463 | family M72 unassigned peptidases | 65.116 | 5.25E-102 | Y | Y | 26 |
| Aa2_1930 | MER0947272 | family S10 unassigned peptidases | 36.047 | 4.77E-09 | Y | N | - |
| Aa2_1967 | MER0333307 | subfamily S26A PA1303 peptidase | 59.162 | 1.54E-78 | Y | N | - |
| Aa2_1979 | MER0923943 | subfamily M16C unassigned peptidases | 50.27 | 1.56E-125 | N | N | - |
| Aa2_1984 | MER1152098 | subfamily M14A unassigned peptidases | 60.372 | 4.17E-141 | Y | Y | 24 |
| Aa2_1991 | MER0991337 | subfamily S9A unassigned peptidases | 74.725 | 2.21E-144 | Y | N | - |
| Aa2_1993 | MER0286455 | family U69 unassigned peptidases | 39.024 | 1.14E-20 | Y | N | - |
| Aa2_1994 | MER0501642 | subfamily M23B unassigned peptidases | 38.053 | 1.4E-11 | N | N | - |
| Aa2_2022 | MER0230219 | subfamily M28F unassigned peptidases | 63.023 | 1.53E-149 | Y | Y | 4 |
| Aa2_2033 | MER0889051 | subfamily M48C unassigned peptidases | 63.303 | 3.2E-100 | Y | N | - |
| Aa2_2052 | MER0511016 | family U32 unassigned peptidases | 53.061 | 1.37E-09 | N | N | - |
| Aa2_2071 | MER0355131 | family S12 unassigned peptidases | 57.895 | 4.46E-77 | Y | N | - |
| Aa2_2106 | MER0040692 | family S33 unassigned peptidases | 38.267 | 2.98E-67 | N | N | - |
| Aa2_2194 | MER0286452 | family U69 unassigned peptidases | 31.982 | 1.71E-08 | N | N | - |
| Aa2_2196 | MER0057986 | family C44 unassigned peptidases | 75.889 | 3.1E-153 | N | N | - |
| Aa2_2299 | MER0209375 | family S33 unassigned peptidases | 46.502 | 6.59E-77 | N | N | - |
| Aa2_2311 | MER0247856 | family C26 At1g63660 | 82.379 | 3.94E-139 | N | N | - |
| Aa2_2323 | MER0224489 | family U32 collagenase | 74.208 | 0 | N | N | - |
| Aa2_2326 | MER1064530 | family S45 unassigned peptidases | 45.64 | 1.41E-164 | N | N | - |
| Aa2_2366 | MER0876950 | family M41 unassigned peptidases | 86.383 | 9.43E-148 | N | N | - |
| Aa2_2372 | MER0441007 | family C26 unassigned peptidases | 76.359 | 0 | N | N | - |
| Aa2_2407 | MER0393879 | family C93 unassigned peptidases | 39.13 | 1.63E-50 | Y | N | - |
| Aa2_2422 | MER0125414 | subfamily S8A unassigned peptidases | 83.911 | 0 | Y | Y | 5 |
| Aa2_2425 | MER0247157 | family M72 unassigned peptidases | 40.435 | 6.42E-43 | Y | Y | 4 |
| Aa2_2475 | MER0092560 | subfamily M50b RseP peptidase | 55.481 | 0 | N | N | - |
| Aa2_2501 | MER0120057 | family M61 unassigned peptidases | 58.29 | 0 | Y | Y | 78 |
| Aa2_2514 | MER0097150 | family S9 unassigned peptidases | 49.412 | 2.01E-56 | N | N | - |
| Aa2_2542 | MER0087690 | family S16 unassigned peptidases | 71.747 | 2.78E-133 | N | N | - |
| Aa2_2553 | MER0087689 | family S11 unassigned peptidases | 76.338 | 0 | Y | N | - |
| Aa2_2577 | MER0206392 | subfamily M28D unassigned peptidases | 60 | 4.03E-99 | Y | N | - |
| Aa2_2584 | MER0115129 | family C26 unassigned peptidases | 73.092 | 8.33E-132 | N | N | - |
| Aa2_2598 | MER0071533 | family S9 unassigned peptidases | 43.162 | 7.53E-69 | N | N | - |
| Aa2_2599 | MER0071533 | family S9 unassigned peptidases | 42.188 | 4.76E-09 | Y | N | - |
| Aa2_2635 | MER0921032 | subfamily M16B unassigned peptidases | 79.29 | 0 | Y | Y | 5 |
| Aa2_2658 | MER1062703 | family S51 unassigned peptidases | 39.427 | 9.09E-59 | Y | N | - |
| Aa2_2699 | MER0469749 | family S12 unassigned peptidases | 46.392 | 1.63E-22 | N | N | - |
| Aa2_2775 | MER0501642 | subfamily M23B unassigned peptidases | 31.959 | 5.31E-17 | N | N | - |
| Aa2_2779 | MER0353939 | family S12 unassigned peptidases | 64.13 | 6.15E-85 | Y | N | - |
| Aa2_2801 | MER0094991 | family C26 unassigned peptidases | 43.519 | 2.06E-58 | N | N | - |
| Aa2_2814 | MER0831538 | family M13 unassigned peptidases | 67.702 | 0 | Y | Y | 5 |
| Aa2_2850 | MER0819368 | subfamily M3A unassigned peptidases | 69.311 | 0 | N | Y | 31 |
| Aa2_2852 | MER0071528 | family M35 EcpA peptidase | 81.818 | 2.97E-96 | Y | Y | 12 |
| Aa2_2853 | MER0478108 | family S9 unassigned peptidases | 61.257 | 8.88E-82 | N | N | - |
| Aa2_2880 | MER0285676 | subfamily S1C unassigned peptidases | 66.867 | 1.09E-153 | Y | N | - |
| Aa2_2881 | MER0407922 | subfamily S1C DegS peptidase | 58.759 | 3.12E-107 | N | N | - |
| Aa2_2902 | MER0087708 | subfamily S8A MCP 01 peptidase | 68.033 | 0 | Y | Y | 59 |
| Aa2_2955 | MER0191870 | subfamily S8A unassigned peptidases | 61.86 | 8.54E-175 | Y | Y | 14 |
| Aa2_2986 | MER0038629 | family M14 unassigned peptidases | 52.871 | 3.16E-158 | Y | N | - |
| Aa2_3071 | MER0501642 | subfamily M23B unassigned peptidases | 30.899 | 7.93E-18 | N | N | - |
| Aa2_3080 | MER0026721 | subfamily S41A unassigned peptidases | 43.438 | 1.01E-84 | Y | N | - |
| Aa2_3091 | MER0191945 | subfamily S8A unassigned peptidases | 54.91 | 4.51E-164 | Y | Y | 15 |
| Aa2_3100 | MER0952361 | subfamily S1A unassigned peptidases | 82.629 | 1.2E-130 | Y | N | - |
| Aa2_3121 | MER0501642 | subfamily M23B unassigned peptidases | 35.233 | 2.38E-19 | N | N | - |
| Aa2_3164 | MER0501642 | subfamily M23B unassigned peptidases | 48.889 | 2.64E-17 | N | N | - |
| Aa2_3165 | MER0501642 | subfamily M23B unassigned peptidases | 50 | 1.03E-19 | N | N | - |
| Aa2_3200 | MER0246142 | subfamily S1C unassigned peptidases | 59.893 | 8.82E-75 | Y | N | - |
| Aa2_3220 | MER1064953 | family S45 unassigned peptidases | 47.232 | 1.55E-176 | N | N | - |
| Aa2_3224 | MER0214273 | family S33 unassigned peptidases | 39.011 | 3.25E-43 | N | N | - |
| Aa2_3284 | MER0815771 | family M1 unassigned peptidases | 74.348 | 9.86E-118 | N | N | - |
| Aa2_3301 | MER0999757 | subfamily S9B unassigned peptidases | 57.563 | 1.35E-98 | N | N | - |
| Aa2_3306 | MER0998060 | subfamily S9B unassigned peptidases | 80.469 | 3.28E-152 | Y | N | - |
| Aa2_3358 | MER0286881 | family M90 MtfA peptidase | 65 | 2.84E-113 | N | N | - |
| Aa2_3361 | MER0501642 | subfamily M23B unassigned peptidases | 33.333 | 1.12E-19 | Y | N | - |

^#^ “Y” means that a protease has a predicted signal peptide or was identified in the secretome, and “N” means that a protease has not a predicted signal peptide or was not identified in the secretome in the table. PSMs, peptide spectrum matches.
